# Nurses’ Expectations of a Knowledge Management System in Nursing Practice: Qualitative Study

**DOI:** 10.2196/78395

**Published:** 2026-01-21

**Authors:** Magdalena Vogt, Sebastian Müller, Glorianna Wagner-Jagfeld, Renate Ranegger, Sabin Zürcher, Janine Vetsch

**Affiliations:** 1 Institute of Health Sciences, Eastern Switzerland University of Applied Sciences, Rosenbergerstrasse 59, St. Gallen, 9000, Switzerland, 41 582571354; 2 Institute of Information and Process Management, Eastern Switzerland University of Applied Sciences, St. Gallen, Switzerland; 3 Department of Research and Development, LEP AG, St. Gallen, Switzerland; 4 Department of Nursing Development, Lindenhofgruppe Bern, Bern, Switzerland

**Keywords:** evidence-based practice, knowledge management, knowledge management system, qualitative research, point of care, nursing

## Abstract

**Background:**

Evidence-based practice is essential for delivering safe, high-quality nursing care; however, its implementation remains challenging due to barriers such as limited knowledge, a lack of supportive organizational culture, and insufficient access to relevant knowledge at the point of care. Knowledge management systems (KMSs) have the potential to bridge this gap by integrating evidence into the nursing process through technological support. Despite growing interest, the integration of KMS into daily nursing practice is still underexplored, especially from the perspective of frontline nurses.

**Objective:**

The aim of this study was to explore nurses’ perspectives on the requirements for a KMS that supports evidence-based practice at the point of care, with a focus on usability, process integration into the electronic nursing care plan and patient chart, and implementation challenges and benefits.

**Methods:**

A qualitative study was conducted in a Swiss hospital using observations, focus groups, and individual interviews with 6 registered nurses, 9 advanced practice nurses, 2 nursing managers, and 1 head physician. Data were analyzed using thematic analysis.

**Results:**

The analysis revealed four main categories and ten subcategories: (1) content of the KMS, (2) personal and structural factors of knowledge management, (3) technical conditions of the KMS, and (4) implementation of a KMS. Participants emphasized the need for an intuitively structured, process-integrated system that links evidence-based information directly to nursing interventions in the electronic nursing care plan and patient chart. Organizational support, interprofessional collaboration, and clear responsibilities were identified as critical for successful implementation.

**Conclusions:**

There is a clear need for a KMS that is user-friendly, seamlessly integrated into clinical workflows, and supports quick, reliable access to evidence-based knowledge. A KMS could enhance nurses’ access to reliable knowledge, promote evidence-based decision-making, and strengthen professional confidence at the point of care. By embedding evidence directly into the electronic nursing care plan and patient chart, such systems can streamline workflows, reduce time spent searching for information, and support more consistent application of best practices. These capabilities may improve information retrieval and contribute to a safer, more consistent nursing practice.

## Introduction

### Background

Delivering safe, high-quality patient care is a central goal of health care institutions [1] and evidence-based practice (EBP) plays a key role in achieving this [2]. Despite strong advocacy, the integration of scientific evidence into everyday nursing practice remains inconsistent [3]. Studies report that barriers such as insufficient EBP knowledge and skills, lack of mentors and facilitators, perceptions that EBP takes too much time, unsupportive organizational cultures, and environments hinder nurses from using evidence at the point of care [2,3]. At the same time, there is an exponential growth in the body of evidence-based knowledge, which needs to be accessed and integrated into daily nursing practices in a timely and contextually relevant manner [4].

To address these challenges, the concept of knowledge management, widely used in other industries, is gaining traction in health care settings [5]. Knowledge management refers to programs or systems to create, capture, store, organize, and share knowledge and information effectively within organizations [6]. In health care settings, knowledge management has the potential to strengthen nursing performance [7] by facilitating access to both scientific knowledge and the expertise or practice knowledge of team members [5]. However, effective knowledge management in nursing practice requires more than just access; it requires integration into clinical workflows, supportive leadership, and a culture of continuous learning [6,8].

Knowledge management systems (KMSs), as a technological solution, offer a way to embed both evidence-based and practice-based knowledge directly into the nursing process [4]. KMSs are designed to support and enhance organizational processes for creating, storing, retrieving, transmitting, and applying knowledge [9]. When effectively designed and implemented, KMSs can support nurses in making informed decisions, promote EBP, and improve the quality of nursing care [9,10]. Despite this potential, research shows that such systems are rarely used in health care, especially in nursing contexts. To date, there are few descriptions of the development, implementation, and evaluation of KMSs in nursing practice [4]. There is a need to investigate factors on the adoption of a KMSs that are integrated into the nursing process in hospitals from different perspectives [11].

### Prior Work and Research Gap

In a prior study, Ranegger et al [12] demonstrated the theoretical feasibility of linking evidence-based knowledge to standardized nursing interventions through a mapping project. While this work provided an essential foundation for embedding evidence in structured nursing documentation, it did not explore how such a system could meet the practical and contextual needs of nurses in clinical settings. Consequently, little is known about what nurses expect from a KMS, how they envision it supporting their workflow, and which organizational factors are required for successful implementation [12].

### Aim of This Study

Building on this gap, our study focuses on advancing current research on KMSs in the health care sector by adding a user-centered perspective to support nurses at the point of care.

Therefore, the aim of this study was to qualitatively explore nurses’ perspectives on the requirements for a KMS that supports EBP at the point of care, with a focus on usability, process integration into the electronic nursing care plan and patient chart, and implementation challenges and benefits. By identifying these requirements, this study contributes to the development of a KMS that is not only theoretically feasible but also contextually relevant, usable, and sustainable in clinical practice.

## Methods

### Study Design

An exploratory qualitative study design based on inductive thematic analysis was conducted to gain an in-depth understanding of nurses’ perspectives, expectations, and experiences related to the development and implementation of a KMS to support EBP at the point of care. The study followed the COREQ (Consolidated Criteria for Reporting Qualitative Research) guidelines to ensure methodological rigor and transparency [13]. The study was underpinned by a pragmatic theoretical orientation, which assumes that knowledge is constructed through experience and that research should focus on understanding real-world problems and generating practical solutions. This framework guided the exploration of nurses’ expectations of a KMS, emphasizing the practical relevance of the findings for system design and implementation.

### Researchers’ Characteristics

Two researchers collected the data. The first researcher was a female research associate with expertise and training in nursing and health sciences. She holds a master’s degree in public health, is specialized in EBP, and has worked as a nurse previously. The second researcher was a male research associate with a master’s degree in information systems with research experience in digital health. The researchers were not known to the participants before the study. Participants were informed about the researchers’ professional backgrounds, institutional affiliations, and the aim of the study. They also knew about the researchers’ roles within the project and that participation was voluntary and anonymous. The researchers were aware that their professional backgrounds could influence how they collected and analyzed data. They therefore reflected these potential biases throughout the analysis to support a balanced understanding of the data.

### Participants and Setting

The study was conducted in a hospital in Switzerland that is part of a private hospital group comprising 3 hospitals. The hospital group employs approximately 2500 staff and treats over 140,000 patients annually, including around 27,000 inpatients. At the time of the study, a new intranet was planned to centralize knowledge resources and improve search capabilities.

The study focused on nurses with diverse work experience and role profiles because the KMS was intended primarily for nursing practice. Additionally, 1 physician was included to provide an interprofessional perspective, as physicians are involved in the current system. Only 1 physician was included because the study primarily focused on nursing workflows and physician involvement in the planned KMS was limited during the recruitment period. A purposive sampling strategy was applied via the head of the nursing development department to recruit participants for observations, individual interviews, and focus group interviews. All participants were directly or indirectly involved in nursing-related knowledge management, either as users of or contributors to knowledge sources. Eligibility criteria included active involvement in nursing care or related managerial or educational functions within the hospital. The initial plan was to invite 15 to 20 participants, which was achieved, with 18 individuals confirming attendance.

### Data Collection

Prior to data collection, an observation guide and a semistructured interview guide were developed based on a literature review. The researchers first conducted independently 4 hours of open, participatory observation on a ward in November 2023, focusing on the activities of 3 registered nurses during their shifts on a surgical and internal medicine ward and took field notes according to the observation guide. These 3 nurses were not further part of the interviews.

Subsequently, all interviews were conducted using the semistructured interview guide, which was adapted after the observations. The 2 authors held 2 face-to-face focus group interviews in the hospital, with participants grouped by professional hierarchy to encourage open discussion. The first focus group involved 9 advanced nurse practitioners (ANPs) with master’s degrees from different wards in the participating hospital (92 min). The second focus group comprised 3 registered nurses with a diploma degree from the surgical ward to capture another perspective (50 min). Three additional online interviews using Microsoft Teams (30 min each) were held with a head physician, a division manager in nursing care, and a co-nursing manager to include different viewpoints. Sociodemographic data from all participants were collected verbally (Table 1). All interviews were conducted between November 2023 and January 2024 and were audio-recorded. Field notes were taken during the interviews. No repeated interviews were carried out.

**Table 1.** Sociodemographic characteristics (N=18).

Sociodemographic characteristics	Value
Sex, female, n (%)	18 (100)
Role, n (%)	
Advanced practice nurse	9 (50)
Registered nurse	6 (33.4)
Nursing management	2 (11.1)
Physician	1 (5.5)
Field of work, n (%)	
Surgical	8 (44.4)
Internal medicine	5 (27.8)
Other (eg, orthopedics and oncology)	3 (16.7)
Expert in a field (eg, delirium and breast care)	2 (11.1)
Years of working experience, n (%)	
<5	1 (5.5)
5-10	7 (38.9)
10-15	5 (27.8)
>15	5 (27.8)

Observation notes were translated, summarized, and thematically clustered. The single and focus group interviews were audio-recorded and transcribed by hand. Data analysis took place in parallel with data collection. Data saturation was considered achieved after the third online interview. Consistency between the 2 data collectors was ensured through continuous discussion during data collection and analysis to align interpretation and maintain reflexivity.

### Data Management and Analysis

Thematic analysis was conducted following the 6-phase approach described by Braun and Clarke [14]. In addition to the categories already formed a priori through a literature review and by developing the semistructured interview guide, 1 author performed the initial coding of all transcripts using MAXQDA 2022 [15]. Coding decisions and theme development were subsequently discussed with 2 additional authors to ensure analytic consistency and to confirm the relevance of identified categories. Disagreements were resolved through discussion until consensus was achieved. The analysis resulted in main categories and subcategories, which were then translated from German to English.

### Ethical Considerations

All participants were informed verbally about the purpose and procedures of the study, data confidentiality, and voluntary participation. Informed consent was obtained before participation, and withdrawal of consent was permitted at any stage, including after data collection. Audio recordings were transcribed verbatim, anonymized to remove any potentially identifiable information, and assigned participant codes before recordings were subsequently deleted. All data were stored securely on password-protected institutional servers in accordance with data protection regulations. No participants withdrew consent for the use of their data in this study. According to Swiss legislation, this study did not require approval by a cantonal ethics committee. In accordance with the Swiss Human Research Act (Humanforschungsgesetz, HFG), ethical approval is mandatory only for research involving human participants where health-related personal data are collected or where interventions are performed [16]. The present study focused exclusively on healthcare professionals’ perspectives on KMSs. No patients were involved, no health-related personal data were collected, and no interventions were performed. Therefore, the study does not fall within the scope of the Swiss Human Research Act and did not require formal ethical approval by a Swiss ethics committee.

However, the study was followed in accordance with the World Medical Association's Declaration of Helsinki.

## Results

### Sociodemographic Characteristics

All 18 participants were nurses with different degrees and roles, except 1 was a head physician. The participants from the observations and interviews had at least 1 year of professional experience and worked in different roles and fields in the hospital (Table 1).

### Categories

The thematic analysis resulted in 4 main categories with 10 subcategories, each of which will be discussed in the following sections (Table 2).

**Table 2.** Main categories and subcategories of the thematic analysis.

Main categories	Subcategories
Content of KMS^a^	Information sources Format of information
Personal and structural factors of knowledge management	Information retrieval skills Time pressure and efficiency
Technical conditions of KMS	Integration into workflow Knowledge access and architecture
Implementation of a KMS	Barriers Facilitators Expected benefits Potential quality indicators

a KMS: knowledge management system.

### Content of KMS

#### Information Sources

Participants described a clear distinction in information sources used by different roles. At the point of care, registered nurses primarily relied on in-house nursing instructions and team members, which was also observed.

In contrast, ANPs accessed a wider range of formal evidence sources, including databases, guidelines, professional networks, and conferences, which they used to update or develop new nursing instructions. Although digital advancements were mentioned, none of the participants reported using artificial intelligence (AI) tools in their knowledge work. Instead, maintaining clear, up-to-date, and evidence-based nursing instructions was viewed as a central way to ensure consistent practice. Most ANPs and nurses from focus groups would support the inclusion of brief synopses of studies explaining changes and evidence updates in the in-house nursing instructions. These would offer nurses an optional, deeper insight into the rationale behind changes. However, some ANPs and the co-nursing manager were critical of this and questioned whether nurses at the point of care would be using this due to the high workload and limited skills in scientific working.

#### Format of Information

Participants acknowledged that the current nursing instructions were logically structured and helpful, often featuring tables of contents and uniform formatting. Nurses were instructed to use nursing standards as the main source of information in nursing practice. At the time point of the interviews and observations, it was therefore important that the nursing standards were written in simple language and regularly updated according to the latest evidence.

Registered nurses and ANPs from both focus groups and observations expressed a need for varied formats as a source of information, such as checklists, videos, and schematics, as long as the content remained concise and practice-oriented. The information in the KMS should not be overloaded and it should summarize the most important information as briefly as possible, as an ANP said:

*I think you have to be careful not to overload nurses with information, to be honest. You have to focus on what you really need in practice. The more it is broken down to the practical situation, the more the knowledge is used*.

[P1]

### Personal and Structural Factors of Knowledge Management

#### Information Retrieval Skills

Participants reported that while they were able to locate nursing instructions within their own specialty, accessing materials outside of their immediate practice area was often time-consuming and frustrating. Nurses, particularly those who were new, part-time, or less experienced, struggled to find information when documents were not intuitively filed or when search paths were long and complex. A nurse confirmed this during the observation. Many participants noted that there was no systematic onboarding to teach information-seeking or navigation strategies. Although some suggested additional training, they emphasized that intuitive structure and powerful search functions were more impactful than teaching workarounds. An ANP summarized it as follows:

*If the search function is poor, it doesn’t matter how well you know the system. You still can’t find what you need.*

[P2]

#### Time Pressure and Efficiency

Time constraints were a significant concern in information use and acquisition across all participants. Nurses commonly relied on team members and ANPs to obtain information quickly, particularly during high workload periods. In the observations, the nurses asked more experienced nurses or a physician in some cases before searching available documents. All participants would find it helpful to have faster access to information sources at the point of care. These sources should be process integrated, which means embedded in the electronic nursing care plan and patient chart. An ANP said:

*I often hear that nurses know that a certain nursing instruction exists. They still ask me as an ANP if I can't just tell them the answer quickly so that they do not have to search for the document*.

[P1]

### Technical Conditions of KMS

#### Integration Into Workflow

Participants envisioned a KMS integrated into every phase of the nursing process, from patient admission and assessment to diagnosis, intervention, and evaluation. They found it important that the information would be available and could be retrieved exactly when they needed it. The nurses from the focus groups saw the greatest benefit in linking information to nursing interventions, for example, to check how a central venous catheter needs to be connected. The head physician also recognized potential in areas like diagnosis support and medication information:

*For example, if I select permanent catheters in the nursing care plan, the relevant nursing instruction should be stored there. If access to the information is clearly visible in the nursing care plan, my attention would be drawn to it and I can just click on it. And then the information just comes up. Because if it is not obvious and I don't see it, I won't click on it and won't get to the information. It has to be obvious to me.*

[P5]

#### Knowledge Access and Architecture

All participants criticized the current dual document storage system, which resulted from an ongoing transition to a new intranet. Most participants found the folder structure confusing and the search function ineffective due to a lack of semantic features. Old or irrelevant documents still appeared in search results, adding to the inefficiency. An ANP mentioned:

*With the folder system, for example, there are folders from the pharmacy, where I think there is a great need for training. Because sometimes you go to an instruction but do not realize that there is also something about [eg,] potassium substitution. And there would be very helpful practical [nursing] instructions. But [most nurses] do not know that they exist.*

[P2]

Suggestions from the participants were to install links in the electronic nursing care plan and patient chart with direct access to information. Two ANPs had the idea to create question mark buttons or to provide the information when clicking on nursing interventions or diagnoses of the electronic nursing care plan and patient chart (eg, dressing a wound, assessing the risk of malnutrition, and administering a medication), which was supported by the other ANPs. Links to documents should always point to the latest version, avoiding discrepancies between sources. An additional idea from the interviewed head physician was to link medication prescriptions directly to the electronic nursing care plan and patient chart with instructions for administration. Additionally, powerful search functions and filter options to quickly find relevant information would be helpful for nurses. The goal from the interviewed division manager in nursing care would be a single-source approach where updated instructions were universally accessible. The division maker in nursing care, therefore, said:

*It must be ensured that the latest version of the nursing instruction is available via the KMS. For example, if you open a link to the nursing instruction from the electronic nursing care plan, the revisions made should also be changed in this document […]. And if something is changed there, I always have the latest version, no matter where I access the document from.*

[P7]

### Implementation of a KMS

#### Barriers

Time, money, and personnel constraints were mentioned as the main barriers to the development and implementation of a KMS. The co-nursing manager stressed that the decision-maker of the hospital needs to be convinced of the KMS, as it requires financial investment. The head nurse emphasized that time and financial resources of the hospital must be used sparingly and that the benefits need to outweigh the costs. Additionally, the lack of clarity around responsibilities for integrating KMS content into hospital IT systems was problematic from the head nurses’ perspective.

#### Facilitators

The ANPs saw themselves as responsible for content conceptualization within the KMS. They proposed that IT staff and KMS providers manage the structural and technical implementation. Strong interprofessional collaboration, clear role descriptions, and leadership support were emphasized as important, as an ANP said:

*The conceptual aspect is for sure with us ANPs. Anything else would be inefficient. But we would not be unhappy if someone else takes care of linking the documents between KMS and the hospital information system.*

[P8]

#### Expected Benefits

Participants believed the KMS would facilitate faster information retrieval, better alignment with current standards, and improved interdisciplinary collaboration. From the head nurse’s point of view, this meant that knowledge in nursing could be better preserved and shared. The nurses were convinced that documents were more likely to be used if they were integrated into the nursing process and could be accessed quickly. This could also increase the nurses’ sense of safety, as they would always use the correct and updated documents. Moreover, the responsibility for finding the right document would no longer lie with the nurses themselves, as a registered nurse said:

*And I think it would be of particular benefit to patients, and that is an interprofessional interest. If the nursing staff can stand up afterwards and say, these are our instructions, we have to implement them. The better you know the content of the nursing instructions and the faster you find them, the better you can argue.*

[P9]

#### Potential Quality Indicators

Participants proposed a range of indicators on how to measure the effectiveness of the KMS. The ANPs mentioned direct KMS-related indicators such as time to retrieve information (eg, reduced time to find nursing instructions), task-completion rate (eg, conducting a nursing intervention), need for help in terms of knowledge retrieval (eg, contacting ANP), and user satisfaction with the system. Indirect quality indicators could be downstream outcomes such as quality of care and patient safety. The nurses from the second focus group mentioned the nurses’ subjective sense of security when performing nursing interventions as an additional indicator. The head physician and co-nursing manager particularly mentioned the quality of the intra- and interdisciplinary communication, including the perceived ease and frequency of collaboration as further quality indicators. The co-nursing manager said:

*For me, relevant indicators are the satisfaction and nurses’ sense of security in their daily work. The nurses need the information to provide the patient with adequate care.*

[P6]

The participants emphasized that an effective KMS should directly support clinical decision-making and increase confidence during care delivery. Nurses frequently linked quick access to correct information with improved performance, lower stress levels, and better patient outcomes. The ANPs and registered nurses believed that evaluating the system’s impact should go beyond technical metrics and include experiential factors, such as how secure, informed, and supported they felt while using the system. Furthermore, participants stressed that if a KMS was truly helpful, it would minimize the need for ad hoc knowledge-seeking from team members, reduce errors, and encourage standardized practice across wards. An ANP mentioned:

*If the information is easy to access whenever they need it, the more they use this information. This, I guess, brings satisfaction because nurses do not have to search a long time for the information and this also indicates a higher sense of security because they know, where they find the information and are well informed.*

[P3]

## Discussion

### Main Results

This study explored nurses’ expectations and needs for a KMS integrated into the electronic nursing care plan and patient chart. Participants found the existing hospital information system fragmented and time-consuming. In-house nursing instructions were well-structured but difficult to access due to a confusing filing system and poor search functionality. Nurses often relied on colleagues or ANPs for quick answers, especially under time pressure. Nurses expressed a strong need for a KMS that was integrated into the electronic nursing care plan and patient chart. They envisioned context-sensitive information access, such as clickable links or icons, at each step of the nursing process, from assessment through intervention to evaluation. The system should offer a simplified structure, powerful search functions, and information presented in practical, user-friendly formats like checklists, videos, or brief summaries. To support safe and efficient care, nurses emphasized that information must be both easily retrievable and always up to date. They saw clarity about responsibilities for maintaining the system as essential. Ultimately, they imagined that a well-designed KMS would enhance care quality, streamline workflows, and strengthen nurses’ professional confidence at the point of care.

### Integrating Knowledge Into Clinical Workflow

Our results show that the current system does not adequately support quick and reliable access to nursing-relevant information at the point of care. Nurses reported relying on team members or navigating complex document systems, often under time pressure. This aligns with findings that emphasize the importance of integrating knowledge tools directly into clinical workflows to reduce search time and cognitive load [4]. Existing help buttons and intranet instructions were appreciated; however, they were not sufficient for efficient knowledge access during daily work. This underscores the importance of embedding knowledge directly into digital workflows. Chorney et al [9] recommended this because they found that integrating KMS into clinical systems significantly improved access and usage. Knowledge embedded in systems not only reduces variation of information and nursing interventions but also supports EBP, given that the content is reliable and up to date [17]. This resonates with the Technology Acceptance Model, which emphasizes perceived usefulness and ease of use as key predictors of usage [18]. The desire for an intuitively designed, workflow-integrated KMS illustrates that these dimensions are central to successful use and implementation.

### Information Literacy and the Role of Training

Nurses described variability in their ability to retrieve and apply information, especially among new staff, part-time workers, or those returning from leave. This reflects a broader challenge of information and digital literacy in nursing practice. Training was seen as critical to ensuring consistent access to and use of available knowledge resources. These findings are consistent with earlier studies that show age and experience influence confidence with electronic clinical systems [10] and that tailored onboarding and continued training support more effective system use [17]. Moreover, nurses’ literacy influences their attitudes towards and intentions to use KMS [19]. While technical solutions are necessary, they must be accompanied by accessible training formats and support structures to ensure equitable use across roles and experience levels [17].

### Evidence Flow and the Role of ANPs

Our study revealed a distinct division of tasks around knowledge sources: while nurses primarily relied on in-house nursing instructions and team members, ANPs engaged with external evidence sources. This distinction reflects the layered process of knowledge use, translation, and transfer outlined by Shahmoradi et al [4]. ANPs acted as translators, adapting external evidence to the hospital’s context, while nurses at the point of care used this adapted knowledge. In addition to the application of nursing instructions, information was also transferred via other communication channels such as direct exchange, emails, or newsletters. This confirms findings from Al-Busaidi [20], who emphasized that knowledge transfer in health care often depends on informal systems that are neither systematic nor easily evaluated.

### KMS Quality, Functionality, and Usability

A consistent theme in the interviews was the desire for a system that was intuitive, accessible, and available throughout the nursing process. This aligns with previous findings that ease of access and integration into clinical routines are critical success factors for KMS adoption [9]. Participants suggested that its functionalities should include a logical filing system, powerful search capabilities, and support for multiple content formats. This reflects a need for information to be both concise and adaptable to diverse learning preferences [9].

The absence of AI use among participants in the period before and during data collection in 2023 and 2024 also reflects broader hesitations in clinical environments. While AI integration was not expected by participants, its future role in enhancing clinical KMS remains a promising area for development [4]. Regardless of the technology used, the success of the KMS depends on its ability to fit seamlessly into the existing workflow and meet users’ needs for quick and trustworthy information [20].

### Evaluation and Trust in the System

Participants proposed a range of indicators to evaluate a future KMS, including efficiency gains, time savings, and perceived improvements in quality of care. These are consistent with indicators described by Al-Busaidi [20], who emphasized both organizational and individual-level outcomes such as improved learning, collaboration, and job satisfaction. Nurses in the interviews also framed evaluation in terms of emotional and ethical relief, particularly the idea that linked and validated instructions could reduce their burden of manually searching the “right” document. This does not imply a reduction in professional responsibility but highlights how a well-maintained KMS can support nurses in fulfilling their responsibilities more safely and confidently. This emotional dimension adds a new perspective in understanding trust in digital systems. Trust is shaped not only by technical reliability but also by how systems redistribute responsibility and reduce the risk of error [11]. When knowledge is institutionalized within a centrally maintained KMS, nurses can rely on the organization rather than the individual for ensuring accuracy. This shift reflects a rebalancing of cognitive and ethical responsibility, which can enhance professional confidence and perceived safety in clinical decision-making [21,22].

### Organizational Conditions for Success

From the point of view of the head physician, division manager in nursing care, and co-nursing manager interviewed, organizational support emerged as an important factor for KMS success. They highlighted the need for leadership support, funding, and clear roles. These themes are confirmed across multiple studies, which identify infrastructure, staffing, policy support, and leadership engagement as critical to implementation success [1,20]. The findings also align with the Normalization Process Theory, which highlights the processes through which new interventions become embedded in everyday practice [23]. The constructs of shared understanding, cognitive participation, and practical integration are evident in participants’ emphasis on collaboration and institutional backing. Organizational culture also plays a key role, as collaborative and open cultures have been found to facilitate KMS adoption more effectively than hierarchical, profit-driven environments [11]. Interviewed ANPs acknowledged the potential value of a KMS, particularly in terms of reducing redundant work and saving time. These findings support the statement from Chorney et al [9], that the success of a KMS is not only a technical or clinical matter, but also a strategic one. For sustainable implementation, the system must align with institutional priorities, demonstrate clear value, and receive long-term support from decision-makers in the setting [20].

### Limitations

The main strength of the study was the inclusion of nurses with different levels of work experience and role profiles. This approach allowed for the consideration of multiple perspectives in the implementation of a KMS that was grounded in practical nursing requirements. The interdisciplinary research team balanced clinical and technical expertise but acknowledged that professional backgrounds might have influenced interpretation. Reflexivity was maintained through ongoing discussion to ensure balanced representation of participants’ views. Conducting the study in a single hospital allowed for detailed observation of local workflows and knowledge management practices but limits the transferability of findings to other settings with different structures or digital maturity. Another limitation concerns the conceptual nature of the topic, as the study explored expectations for a KMS that has not yet been developed. Finally, the data were collected in German and then translated into English. These translations were rigorously checked by authors fluent in both languages.

### Implications for Nursing Practice and Research

Our findings underscore the importance of designing a KMS that supports nurses’ real-time information needs at the point of care. Seamless integration into the electronic nursing care plan and patient chart, intuitive navigation, and access to up-to-date, evidence-based instructions in various formats were seen as essential. Nurse managers should prioritize training, onboarding processes, and continuous support, especially for new, part-time, or returning staff.

There is a need for further research on the design and usability of KMS tools, especially those that leverage emerging technologies such as AI for knowledge synthesis and decision support. Future studies should also explore the implementation and effects of KMS at the point of care. Further investigation into the quality indicators identified by nurses for measuring KMS impact could support the development of validated evaluation frameworks. Future projects from the authors focus on developing and piloting an AI-supported KMS. It aims to provide personalized, evidence-based recommendations tailored to nurses’ skill levels and workflows, thereby enhancing safety, quality, and efficiency at the point of care.

### Conclusions

Participants expressed a clear need for a KMS that is user-friendly, seamlessly integrated into clinical workflows, and supports quick, reliable access to evidence-based knowledge. A well-designed KMS may have the potential to not only improve care quality and efficiency but also to enhance nurses’ confidence and sense of safety in their daily work.

## Abbreviations

AI: artificial intelligence
ANP: advanced nurse practitioner
COREQ: Consolidated Criteria for Reporting Qualitative Research
EBP: evidence-based practice
KMS: knowledge management system
